# Do Pain and Autonomic Regulation Share a Common Central Compensatory Pathway? A Meta-Analysis of HRV Metrics in Pain Trials

**DOI:** 10.3390/neurosci6030062

**Published:** 2025-07-05

**Authors:** Marianna Daibes, Bassel Almarie, Maria Fernanda Andrade, Giovanna de Paula Vidigal, Nadine Aranis, Anna Gianlorenco, Carlos Bandeira de Mello Monteiro, Prateek Grover, David Sparrow, Felipe Fregni

**Affiliations:** 1 Neuromodulation Center and Center for Clinical Research Learning, Spaulding Rehabilitation Hospital and Massachusetts General Hospital, Harvard Medical School, Boston, MA 02138, USA; mariannadaibesr@gmail.com (M.D.); mariafernandaandrade12@gmail.com (M.F.A.); naranis@mghihp.edu (N.A.); gianlorenco@ufscar.br (A.G.); 2 Department of Physical Therapy, Speech and Occupational Therapy, Faculty of Medicine, University of São Paulo (USP), Sao Paulo 05508-220, Brazil; carlosfisi@uol.com.br; 3 Department of Physical Therapy, Federal University of Sao Carlos, Sao Paulo 13565-905, Brazil; 4 Limb Loss, Difference, and Preservation Rehabilitation Center for Research, Education, and Service (LimbRehabCaRES), Penn State College of Medicine, Hershey, PA 17033, USA; pgrover@pennstatehealth.psu.edu; 5 Department of Medicine, Boston University Chobanian and Avedisian School of Medicine, and VA Normative Aging Study, Veterans Affairs Boston Healthcare System, Boston, MA 02118, USA; david.sparrow@va.gov

**Keywords:** heart rate variability, chronic pain, autonomic nervous system, systemic inflammation

## Abstract

Background: Chronic pain is closely associated with dysregulation of the autonomic nervous system, often reflected by reduced heart rate variability (HRV). While observational studies have demonstrated this association, the extent to which pain interventions modulate HRV and the impact of individual factors on HRV changes remain unclear. Objective: To evaluate the impact of pain interventions on HRV parameters through meta-analysis of randomized controlled trials (RCTs), and to examine whether intervention type and individual factors such as body mass index (BMI) moderate HRV responses. Methods: We conducted a systematic review of 23 RCTs and a meta-analysis of 21 RCTs (1262 subjects) involving patients with acute and chronic pain. HRV outcomes were extracted pre- and post-intervention. Both between-group (active vs. sham/control) and one-group (pre-post within active group) analyses were performed for time-domain indices—standard deviation of normal-to-normal intervals (SDNN), root mean square of successive differences (RMSSD), and percentage of successive normal-to-normal intervals >50 ms (pNN50)—and frequency-domain indices—high-frequency (HF) and low-frequency (LF) components. Meta-regressions tested moderators including BMI, age, and pain phenotype. The protocol was registered in PROSPERO (CRD42023448264). Results: Twenty-three RCTs involving 1262 participants with a wide range of pain conditions were included. Meta-analysis of time-domain HRV parameters showed a trend toward improvement: SDNN (g = 0.435, *p* = 0.059) approached significance, while RMSSD (g = 0.361, *p* = 0.099) and pNN50 (g = 0.222, *p* = 0.548) showed smaller, non-significant effects. Frequency-domain analysis revealed a significant moderate reduction in the LF/HF ratio (g = −0.378, *p* = 0.003), suggesting a shift toward parasympathetic dominance. HF and LF showed small, non-significant changes. One-group meta-analysis confirmed significant improvements in vagally mediated HRV, with large effects for RMSSD (g = 1.084, *p* < 0.001) and HF (g = 0.622, *p* < 0.001), and a moderate effect for SDNN (g = 0.455, *p* = 0.004). Meta-regression identified BMI as a significant moderator: higher BMI was associated with attenuated improvements in HF and RMSSD and a slight shift toward sympathetic predominance. **Conclusions:** Pain interventions can significantly modulate autonomic function, as reflected in HRV improvements, particularly in vagally mediated indices. These effects are influenced by patient characteristics such as BMI. HRV may serve as a valuable biomarker for both treatment efficacy and autonomic recovery in pain management. In this context, HRV highlights its role as a biomarker for pain dysregulation and compensatory failure, reflecting shared top-down modulation between nociception and autonomic regulation.

## 1. Introduction

Pain imposes a substantial burden on health and longevity [1,2]. Among individuals under 50 years of age, chronic pain is associated with a 2.5- to 3-fold increase in mortality compared to the general population, underscoring the serious and often underrecognized consequences of persistent pain [3,4]. In diseases characterized by pain involving both central and peripheral mechanisms—such as systemic lupus erythematosus, rheumatoid arthritis (RA), and fibromyalgia [5,6,7]—autonomic nervous system (ANS) dysfunction, reflected by reduced heart rate variability (HRV), has been consistently observed. HRV indices can be categorized into time-domain and frequency-domain, and non-linear categories.

Importantly, this autonomic imbalance, represented by HRV, appears to be linked more directly to inflammation than to pain intensity alone. For instance, in patients with systemic lupus erythematosus, lower HRV indices—particularly sample entropy—were inversely associated with levels of inflammatory biomarkers such as high-sensitivity C-reactive protein (hsCRP) and myeloperoxidase, suggesting a relationship between systemic inflammation and impaired cardiac autonomic control, independent of disease activity or pain [8,9]. Supporting this, Koopman et al. (2016) demonstrated in both preclinical and clinical studies that autonomic dysregulation precedes rheumatoid arthritis onset and is predictive of disease development [10]. In rheumatoid arthritis patients, reduced parasympathetic tone, as measured by HRV, was correlated with higher levels of inflammatory cytokines like tumor necrosis factor-alpha (TNF-α) and interleukin-6 (IL-6). Moreover, individuals with higher vagal tone responded better to anti-TNF therapies, indicating that autonomic balance may modulate inflammatory responses [11,12]. Similarly, imaging and physiological studies in patients with chronic inflammatory joint diseases have highlighted the role of the insular cortex—a brain region implicated in both pain perception and autonomic regulation. Alterations in insular cortex volume correlated with heart rate changes and were proposed as neural correlates of dysregulated autonomic nervous system function in chronic pain states [13].

These insights support the hypothesis that autonomic abnormalities (e.g., reduced HRV) and chronic pain may not be causally linear, but instead parallel manifestations of a shared deregulatory process involving immune signaling, central nervous system plasticity, and impaired vagal tone [14,15]. Observational studies find lower HRV in chronic pain, but intervention trials are inconsistent, often ignore HF and LF/HF, and rarely test moderators such as BMI or pain phenotype [16,17]. Given the evidence that inflammation-driven neural and microvascular injury contributes to both persistent pain and autonomic dysfunction [18,19], we aim to systematically evaluate whether clinical interventions that alleviate pain also restore autonomic balance, as reflected in HRV metrics. Therefore, the objective of this meta-analysis is to assess randomized controlled trials of pain interventions to answer the following key questions: (1) Do HRV parameters significantly differ between active and sham treatment groups? (2) Are changes in HRV more pronounced when evaluating pre-post effects within the active treatment arm, thereby capturing the full effect of the intervention—including any placebo component? (3) Are specific types of interventions—such as neuromodulation, pharmacologic, or physical therapies—more likely to impact HRV? Finally, (4) are these autonomic effects moderated by markers of systemic inflammation, such as body mass index (BMI), which could serve as surrogates for inflammatory load and individual susceptibility to autonomic dysregulation?

## 2. Methods

### 2.1. Protocol and Registration

This systematic review was conducted according to the PRISMA statement (Preferred Reporting Items for Systematic Review and Meta-Analyses) [20]. The protocol was registered a priori in PROSPERO (registration number CRD42023448264).

### 2.2. Search Strategy

The searches were conducted in PubMed, CENTRAL, and Embase databases in July 2023 and updated in March 2025. The main keywords for the search strategy were “AUTONOMIC NERVOUS SYSTEM” OR “ANS” OR “HEART RATE VARIABILITY” OR “HRV” AND “PAIN”. The search strategy was built by combining free-text and MeSH terms and combined using the Boolean operator OR. Between the components AND was used as Boolean operator. No additional search filters were used in this study. We also screened the reference lists of all relevant publications for additional papers (backward reference search).

### 2.3. Eligibility Criteria

All randomized controlled clinical trials (RCTs) reporting any HRV parameters as an outcome on patients older than 18 years with any pain condition (i.e., acute or chronic) published in English were included. Non-RCTs, observational studies, reviews, commentaries, and editorials were excluded.

### 2.4. Data Collection and Extraction

**Figure 1:**
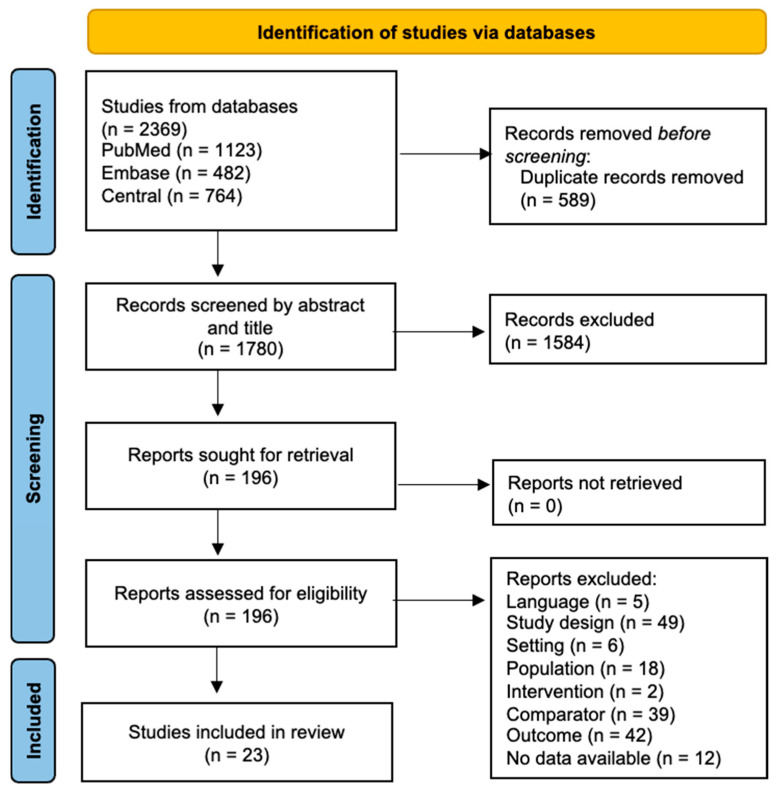
PRISMA flow of study selection.

Search results were screened using Covidence online software tool (Melbourne, Veritas Health Innovation, Australia). After removing duplicate articles, the title and abstract were screened by four separate reviewers independently (MD, GV, MA, and BA). Subsequently, two reviewers performed the full-text screening independently from each other (MD and GV). Discrepancies were discussed after each stage, and consensus was achieved by an independent reviewer (MA). Finally, 23 studies were included in the review, reported in Figure 1.

Data extraction was performed in an Excel template, with the following items: (1) bibliometric variables, author(s), year of publication, and country; (2) population variables, sample size, sex, age, BMI, and diagnosis; (3) intervention; and (4) outcome measures. The participant and intervention characteristics of the included RCTs are reported in Table 1.

#### 2.4.1. Intervention Measure

A meta-analysis was performed, grouping studies with similar association variables and comparable intervention measures. In this context, (a) pain duration (i.e., acute or chronic), and (b) pain intensity at baseline and post-intervention were considered to perform sub-group analysis. The final decision was made post hoc, although the plan to pool comparable data was outlined ‘a priori’ in our study protocol.

#### 2.4.2. Outcome Measures

Following the Task Force of the European Society of Cardiology and the North American Society of Pacing and Electrophysiology in 1996 [21], we aimed to explore the changes in HRV metrics in patients with pain following different types of intervention.

HRV variables consist of time-domain and frequency-domain indices. The time-domain variables extracted were (a) Root Mean Square of Successive R-R interval Differences (RMSSD), (b) Standard Deviation of N-N intervals (SDNN), and (c) percentage of successive normal-to-normal intervals which exceed 50 ms from the previous one (pNN50). Frequency-domain components considered were (a) low-frequency (LF), (b) high-frequency (HF), and (c) low-frequency/high-frequency ratio. The LF component reflects heart rate variations within a frequency span of 0.04 to 0.15 Hz, often interpreted as predominantly reflecting sympathetic nervous system activity. On the other hand, the HF component, spanning a frequency range from 0.15 to 0.40 Hz, is considered an indicator of parasympathetic nervous system influence. The ratio of these two parameters describes the sympathovagal balance [22,23].

**Table 1:** Participant and intervention characteristics of the included RCT studies (n = 23).

Author, Year	Country	N Subjects (Interv/Total)	Study Design	Age *	Female (%)	BMI (kg/m^2^) *	Diagnosis	Intervention	Comparator	Type of Pain (Duration)	Pain Scale	Assessment Period
Bácker M, 2008 [24]	Germany	9/19	Parallel	43.5 ± 8.5	15 (88.2)	n/r	Migraine	Acupuncture	Sham	Acute	PDI	Baseline and 12 weeks
Berry M, 2014 § [25]	USA	8/14	Pilot	41.8 ± 6.6	1 (12)	n/r	Chronic pain	Biofeedback	No intervention	Chronic	BPI	Baseline and 4 weeks
Buttagat V, 2021 [26]	Thailand	18/36	Parallel	22.9 ± 3.4	11 (61.11)	20.3	Back pain associated with myofascial pain syndrome	Thai massage	No intervention	Chronic	VAS	Baseline and immediate post-intervention
Cerritelli F, 2021 [27]	Italy	15/30	Parallel	42.3 ± 7.3	7 (40)	24.1 ± 3.5	Low back pain	Osteopathic manipulative treatment	Sham	Chronic	VAS	Baseline, immediate (T1), and sustained response (T2), 4 wks.
Espejo-Antúnez L, 2021 [28]	Switzerland	25/49	Parallel	37.0 ± 6.6	0 (0)	25.21 ± 2.8	Low back pain	Interferential current therapy	Sham	Chronic	NPRS	Baseline and Immediate effect
Fernández-Morales C, 2024 § [29]	Spain	14/31	Pilot	24.0 ± 5.5	n/r	24.0 ± 1.4	Flight-related neck pain	Physical therapy	No intervention	Acute	NPRS	Baseline and 4 wks, 8 sessions
Hu X, 2021 [30]	China	50/100	Parallel	50.8 ± 15.3	28 (56)	24.8 ± 2.4	Chronic non-specific low back pain (CNSLBP)	Silver needle therapy	No intervention	Chronic	NRS	Baseline, 1, 2, 3, and 6 months
Jiang W, 2011 [31]	USA	20/40	Parallel	55.1 ± 14.4	9 (45)	31.00 ± 8.1	Painful diabetic neuropathy	Pregabalin	Placebo	Chronic	VAS	Baseline and 4 wks
Liao CD, 2017 [32]	Taiwan	22/44	Parallel	50.78 ± 8.08	17 (77.3)	21.96 ± 2.31	Neuropathic pain	Stellate ganglion irradiation	Sham	Chronic	VAS	Baseline and 6 wks, 12 sessions
Matsubara T, 2011 * [33]	Japan	11/33	Parallel	35.5 ± 6.4	11 (100%)	n/r	Neck Pain	Local acupuncture	No intervention	Chronic	VRS	Baseline and Immediate effect
Matsubara T, 2011 * [33]	Japan	11/33	Parallel	37.2 ± 7.0	11 (100%)	n/r	Neck Pain	Distal acupuncture	No intervention	Chronic	VRS	Baseline and Immediate effect
Moreira RM, 2023 § [34]	Brazil	9/18	Pilot	58 ± 9.89	9 (100)	28.27 ± 3.54	Fibromyalgia	Acupuncture	No intervention	Chronic	NPRS	Baseline and 6 wks, 6 sessions
Olliges E, 2024 * [35]	Germany	21/60	Parallel	64.19 ± 9.3	12 (42.8)	n/r	Knee Osteoarthritis	OLP-pain	No intervention	Chronic	NRS	Baseline and 3 weeks
Olliges E, 2024 * [35]	Germany	20/60	Parallel	66.8 ± 9.7	9 (45)	n/r	Knee Osteoarthritis	OLP-mood	No intervention	Chronic	NRS	Baseline and 3 weeks
Paccione CE, 2022 * [36]	France	28/116	Parallel	48.25 ± 8.88	27 (96.4)	29.26 ± 6.49	Fibromyalgia	Active tVNS	Sham	Chronic	NRS	Baseline and 2 weeks
Paccione CE, 2022 * [36]	France	29/116	Parallel	48.25 ± 8.88	27 (96.4)	29.26 ± 6.49	Fibromyalgia	Active MDB	Sham	Chronic	NRS	Baseline and 2 weeks
Patel ABU, 2023 [37]	UK	43/86	Parallel	47 (33 to 54)	17 (39.6)	n/r	Perioperative pain after orthopedic surgery	Transcutaneous auricular nerve stimulation	Sham	Acute	VAS	Baseline (before surgery) and After surgery
Paz T, 2023 * [38]	Brazil	20/58	Parallel	40 ± 17	15 (75)	n/r	Musculoskeletal pain	Spinal Manipulation	Placebo	Chronic	NRS	Baseline and Immediate effect
Paz T, 2023 * [38]	Brazil	19/58	Parallel	48 ± 11	13 (68)	n/r	Musculoskeletal pain	Myofascial Manipulation	Placebo	Chronic	NRS	Baseline and Immediate effect
Prim JH, 2019 †,§ [39]	USA	20/20	Pilot-Crossover	43 ± 13.37	12 (60)	25.94 ± 4.46	CLBP	Transcranial alternating current stimulation	Placebo	Chronic	DVPRS	Baseline and Immediate effect
Toro-Velasco C, 2009 † [40]	Spain	11/11	Crossover	51 ± 15	8 (60)	n/r	CTTH	Head-neck massage	Placebo	Chronic	NPRS	Baseline, Immediate and after 24 h, 2 sessions
Warth M, 2015 [41]	Germany	42/42	Parallel	63.8 ± 14.1	28 (66.7)	n/r	Palliative care pain	Music therapy	No intervention	Acute	VAS	Baseline and at 48 h after 2 sessions.
Wong A, 2017 [42]	Korea	17/31	Parallel	51 ± 2	18 (100)	23.1 ± 0.5	Fibromyalgia	Tai Chi	No intervention	Chronic	VAS	Baseline and 12 weeks
Xuan J, 2022 [43]	China	21/42	Parallel	51.14 ± 8.66	7 (33.3)	25.07 ± 3.77	Pancreatitis pain	Transcutaneous electrical acustimulation	Sham	Acute	VAS	Baseline and 48 h
Yamamoto K, 2011 § [44]	Japan	9/18	Pilot	n/r	3 (33)	n/r	Incurable stomach cancer during palliative care for pain	WW-Footbath Method	No intervention	Chronic	VAS	Baseline and Immediate effect
Yeh ML, 2018 [45]	Taiwan	39/80	Parallel	55.64 ± 14.83	24 (61.5)	n/r	Post-operative pain in hemorrhoidectomy	Transcutaneous acupoint electrical stimulation	No intervention	Acute	VAS	Baseline and after 4 sessions
Younes M, 2017 [46]	France	10/17	Parallel	31 ± 9	0 (0)	23.67 ± 4.34	Low back pain	Spinal manipulative treatment	Sham	Acute	NPRS	Baseline and immediate effect, 2 sessions

* Matsubara T, 2011; Paccione CE, 2022; Paz T, 2023 and Olliges E, 2024 are parallel RCTs with three or more groups. † Toro-Velasco C, 2009; Prim JH, 2019; are crossover RCTs. § Yamamoto K, 2011; Prim JH, 2019; Moreira RM, 2023; Fernández-Morales C, 2024 and Berry, 2014 are pilot RCTs. * Bácker, 2008 demographics include entire sample values. * Age and BMI are reported as mean + SD, except for Patel ABU, 2023, which is in range. Pain was assessed in all articles at baseline. Abbreviations: BPI: Brief Pain Inventory; CLBP: Chronic low back pain; CTTH: Chronic tension-type headache; DVPRS: Defense and Veterans Pain Rating Scale; MDB: Meditative-Based Diaphragmatic Breathing; NPRS: Numerical Pain Rating Scale; NRS: Numeric Rating Scale; PDI: Pain Disability Index; tVNS: Transcutaneous Vagus Nerve Stimulation; VAS: Visual Analog Scale; VRS: Verbal rating scale.

All parameters were extracted at baseline and post-intervention. Studies are reported with the metrics that were presented in the original studies, meaning no calculations were performed to obtain standardized metrics. The recording methods for HRV measurements were also registered for further discussion (device, instructions, preparation, position, collection time, analysis software, and analysis time).

### 2.5. Risk of Bias Assessment

The risk of bias was evaluated using the Cochrane Collaboration’s tool (Risk of Bias Tool 2.0). Included RCTs were analyzed across five domains: the randomization process (D1), deviations from the intended intervention (D2), missing outcome data (D3), outcome measurement (D4), and selective reporting of results (D5). Each domain was graded as low risk (low), moderate risk (some concerns), or high risk (high) by two assessors independently (MA and NA). A final score of bias was determined after the five domains were evaluated. Discrepancies were resolved through consensus. Studies were not excluded based on the results of the risk of bias assessment to avoid the selective reporting of study findings.

### 2.6. Heterogeneity Assessment

Heterogeneity among studies was assessed using Cochran’s Q test and the I^2^ statistic. A *p*-value < 0.05 on Cochran’s Q test was considered statistically significant heterogeneity, while I^2^ values were interpreted as follows: <30% as low, 30–50% as moderate, and >50% as high heterogeneity. Publication bias was examined using funnel plots and Egger’s test, with a *p*-value < 0.05 suggesting small publication bias.

### 2.7. Data Analysis

The effect size was calculated as the mean difference of HRV metrics between the intervention and control groups at baseline versus post-intervention, using Hedges’ g statistic. The statistical difference in the pooled effect size was computed using a 95% confidence interval (CI). A random-effects model was adapted to account for anticipated heterogeneity, providing a more conservative estimate of the effect size than the fixed model. In addition to between-group comparisons, we conducted one-group meta-analyses using pre- and post-intervention data from the active treatment arms to capture the total effect of interventions. For each included study, we extracted the means and standard deviations of HRV parameters before and after intervention. This analytic strategy allowed us to assess the full magnitude of autonomic modulation following pain interventions. Meta-regression analyses were conducted to explore the moderating effect of BMI on the association between interventions and HRV outcomes. Separate mixed-effects meta-regression models were fitted for each HRV parameter, with BMI included as a continuous moderator. Regression coefficients, standard errors, and *p*-values were calculated, along with the proportion of explained variance (R^2^) and residual heterogeneity (I^2^). In addition to assessing BMI as a main effect, moderator analyses were performed using the Q-model test to determine whether BMI significantly modified intervention effects. Data were synthesized and analyzed using the software R Statistical Software (version 4.2.3; R Core Team, 2024).

## 3. Results

### 3.1. Study Selection

Twenty-three RCTs were included with a total sample size of 1262 subjects [24,25,26,27,28,29,30,31,32,33,34,35,36,37,38,39,40,41,42,43,44,45,46]. The mean age of the overall population was 44.3 years (SD = 10.4). Women comprised 52.6% of the study subjects. Diagnoses treated in the trials included migraine, chronic neck pain, low back pain, fibromyalgia, osteoarthritis, perioperative pain, post-operative hemorrhoidectomy, acute mechanical back pain, neuropathic pain (including complex regional pain syndrome type 1, postherpetic neuralgia, and post-surgery pain), painful diabetic neuropathy, chronic non-specific low back pain, acute pancreatitis, musculoskeletal pain, palliative pain, chronic unspecified pain, chronic tension-type headache, and back pain associated with myofascial pain syndrome. HRV was evaluated in all studies both at baseline and after pain interventions, with a recording session duration that had a median of 5 min (IQR: 5 to 10 min), ranging from 2 to 60 min. Table 2 describes HRV variables and recording methods considered in the included studies.

### 3.2. Time-Domain Measures

**Figure 2:**
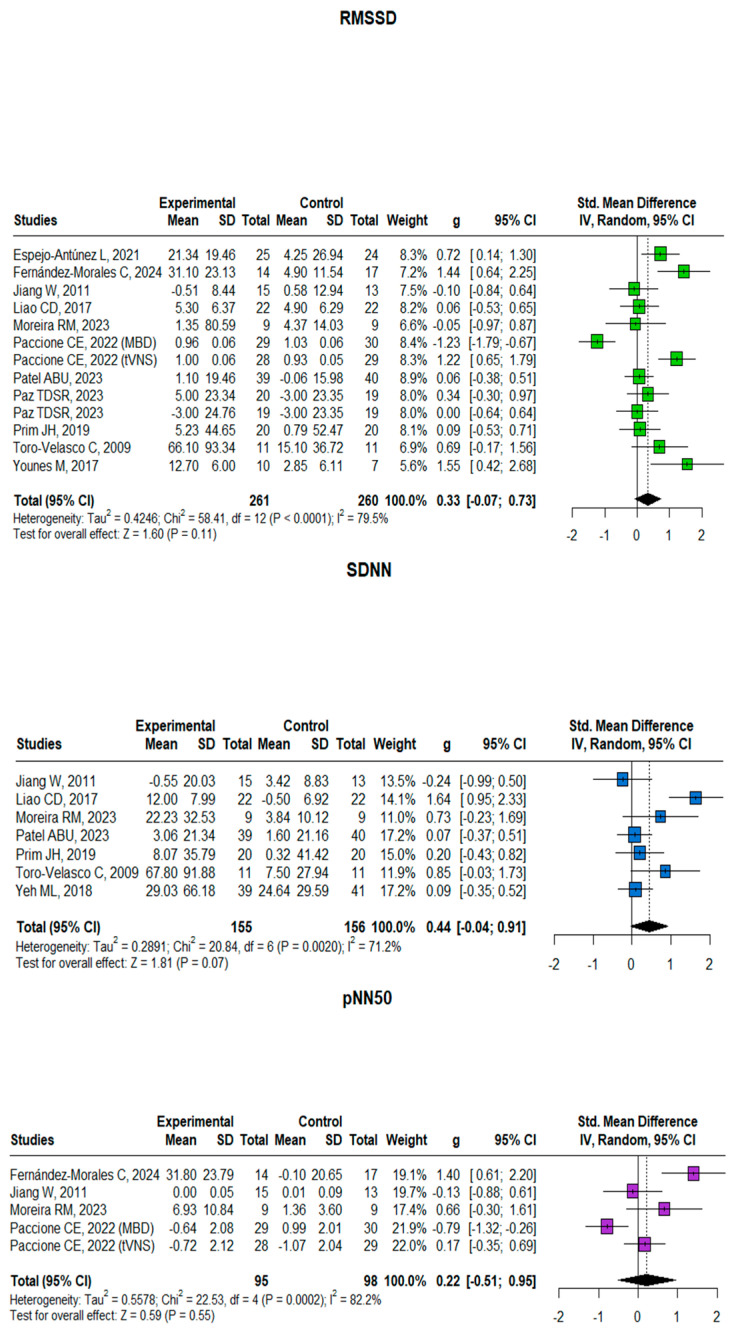
Forest plot for RMSSD, SDNN, and pNN50 comparing experimental and control groups. The plots show standardized mean differences (SMDs) with 95% confidence intervals. Included studies for SDNN: Espejo-Antúnez et al. [28], Fernández-Morales et al. [29], Jiang et al. [31], Liao et al. [32], Moreira et al. [34], Paccione et al. [36], Patel et al. [37], Paz et al. [38], Prim et al. [39], Toro-Velasco et al. [40], Younes et al. [46]. Included studies for pNN50: Jiang et al. [31], Liao et al. [32], Moreira et al. [34], Patel et al. [37], Prim et al. [39], Toro-Velasco et al. [40], Yeh et al. [45]. Included studies for RMSSD: Fernández-Morales et al. [29], Jiang et al. [31], Moreira et al. [34], Paccione et al. [36].

The meta-analyses of HRV parameters revealed varying effects across the three time-domain measures (Figure 2). For RMSSD (13 studies, n = 521), the analysis showed a small-to-moderate positive effect (Hedges’ g = 0.33, 95% CI [−0.077, 0.730], *p* = 0.11) with substantial heterogeneity (I^2^ = 79.5%). SDNN analysis (7 studies, n = 311) demonstrated a moderate positive effect approaching significance (Hedges’ g = 0.44, 95% CI [−0.04, 0.91], *p* = 0.07) with considerable heterogeneity (I^2^ = 71.2%). The pNN50 analysis (5 studies, n = 193) yielded the smallest effect size (Hedges’ g = 0.22, 95% CI [−0.51, 0.95], *p* = 0.55) with substantial heterogeneity (I^2^ = 82.2%). While all three parameters showed positive effect sizes, none reached statistical significance at the conventional *p* < 0.05 level, with SDNN showing the strongest trend toward significance. All parameters demonstrated substantial between-study heterogeneity (I^2^ > 70%), suggesting considerable variation in intervention effects across studies.

### 3.3. Frequency-Domain Measures

**Figure 3:**
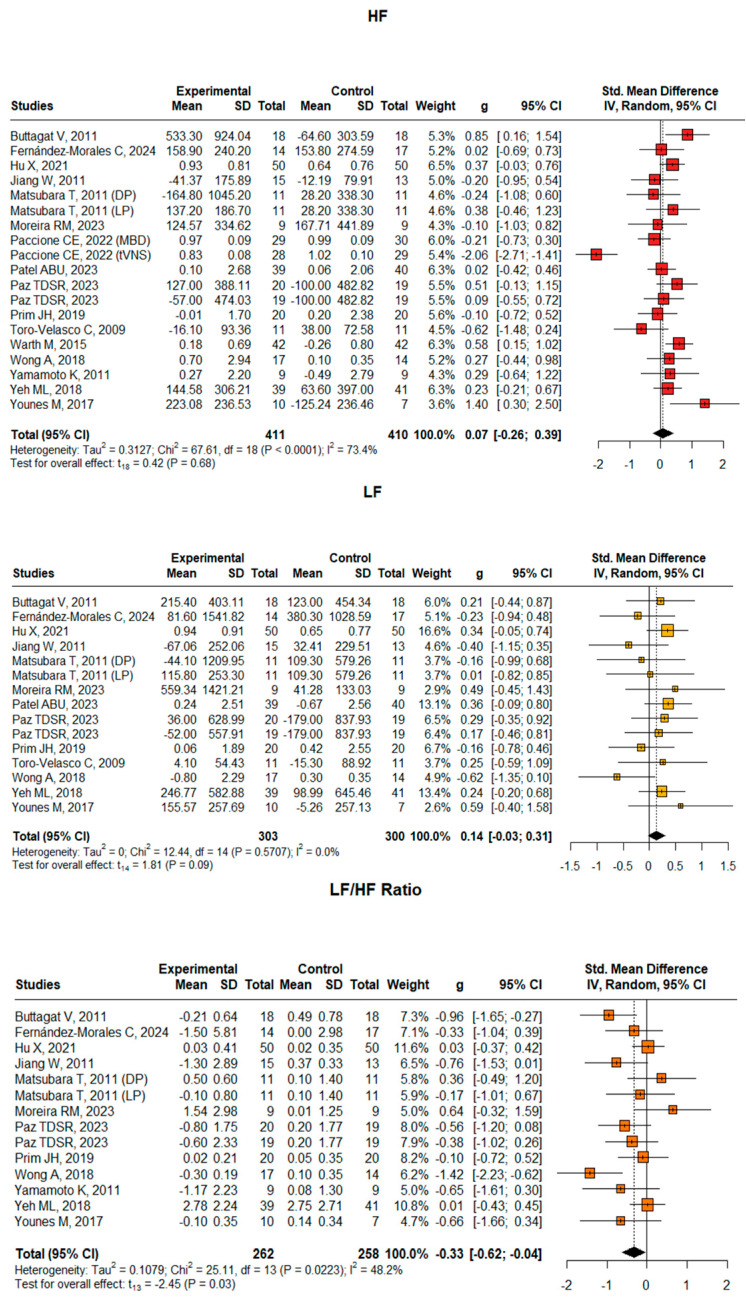
Forest plot for HF, LF, and LF/HF ratio comparing experimental and control groups. The plots show standardized mean differences (SMDs) with 95% confidence intervals. Included studies for LF: Buttagat et al. [26], Fernández-Morales et al. [29], Hu et al. [30], Jiang et al. [31], Matsubara et al. [33], Moreira et al. [34], Paccione et al. [36], Patel et al. [37], Paz et al. [38], Prim et al. [39], Toro-Velasco et al. [40], Warth et al. [41], Wong et al. [42], Yeh et al. [45], Younes et al. [46]. Included studies for LF/HF ratio: Buttagat et al. [26], Fernández-Morales et al. [29], Hu et al. [30], Jiang et al. [31], Matsubara et al. [33], Moreira et al. [34], Paccione et al. [36], Patel et al. [37], Paz et al. [38], Prim et al. [39], Toro-Velasco et al. [40], Yeh et al. [45], Younes et al. [46]. Included studies for HF: Buttagat et al. [26], Fernández-Morales et al. [29], Hu et al. [30], Jiang et al. [31], Matsubara et al. [33], Moreira et al. [34], Paccione et al. [36], Toro-Velasco et al. [40], Yeh et al. [45], Younes et al. [46].

Based on the meta-analyses of frequency-domain HRV parameters, differential effects were observed across the three measures (Figure 3). LF power showed a small positive effect (Hedges’ g = 0.14, 95% CI [−0.03, 0.31], *p* = 0.09) with notably consistent findings across studies (I^2^ = 0%). HF power demonstrated the smallest effect size (Hedges’ g = 0.07, 95% CI [−0.26, 0.39], *p* = 0.68) but exhibited substantial heterogeneity between studies (I^2^ = 73.4%). Most notably, the LF/HF ratio showed a significant moderate negative effect (Hedges’ g = −0.33, 95% CI [−0.62, −0.004], *p* = 0.03) with moderate heterogeneity (I^2^ = 48.2%). This significant reduction in the LF/HF ratio, combined with the non-significant trends in individual LF and HF components, suggests a potential shift in autonomic balance following intervention, possibly indicating a relative increase in parasympathetic modulation compared to sympathetic activity. The analysis encompassed a substantial dataset (LF: 15 studies, n = 603; HF: 19 studies, n = 821; LF/HF: 14 studies, n = 520), providing robust evidence, particularly for the LF/HF ratio findings, while the heterogeneity in HF power suggests variable intervention effects across different study contexts.

### 3.4. One-Group Meta-Analysis (The Combined Effect of the Intervention)

The one-group meta-analysis of HRV parameters revealed significant improvements in vagal and overall HRV following intervention. RMSSD demonstrated a large and statistically significant pooled effect size (Hedges’ g = 1.084, *p* < 0.001), indicating robust increases in vagal-mediated HRV. HF power also showed a significant positive effect (g = 0.622, *p* < 0.001), further supporting enhanced parasympathetic activity. SDNN exhibited a moderate, significant effect (g = 0.455, *p* = 0.004), while pNN50 (g = 0.186, *p* = 0.469) and LF (g = 0.099, *p* = 0.447) showed small, non-significant changes. The LF/HF ratio did not show a significant change (g = −0.087, *p* = 0.581). Heterogeneity was high across most parameters, suggesting substantial variability between studies.

### 3.5. Meta-Regression

The meta-regression analyses revealed differential effects of BMI on HRV parameters, with the strongest relationships observed for HF power and RMSSD. As a main effect, BMI demonstrated a negative association with HF power (coefficient = −0.172, SE = 0.088, *p* = 0.108), explaining 43.3% of the variance in effect sizes (R^2^ = 0.433), indicating that individuals with higher BMI showed reduced HF power responses. Similarly, RMSSD showed a negative relationship with BMI (coefficient = −0.158, SE = 0.187, *p* = 0.445, R^2^ = 0.152), suggesting that higher BMI values were associated with reduced vagally mediated HRV responses. When tested as a moderator, BMI significantly modulated the intervention effects on HF power (Q_model = 22.286, *p* < 0.001) and RMSSD (Q_model = 7.220, *p* = 0.007), with substantial residual heterogeneity (I^2^ = 82.89% and 90.09%, respectively). The LF/HF ratio showed a weak positive moderation effect (coefficient = 0.047, SE = 0.069, *p* = 0.541, R^2^ = 0.136), suggesting slightly higher sympathovagal balance in those with elevated BMI, while LF power demonstrated a slight negative association (coefficient = −0.047, SE = 0.055, *p* = 0.458, R^2^ = 0.194).

These findings suggest that BMI plays a more pronounced role in modulating vagal-related HRV parameters (HF power and RMSSD) compared to sympathetically mediated or balanced measures (LF power and LF/HF ratio), with higher BMI generally associated with attenuated HRV responses to interventions. Specifically, as BMI increases, there is a consistent pattern of reduced vagal tone (lower HF power and RMSSD) and a slight shift toward sympathetic predominance (higher LF/HF ratio), suggesting that elevated BMI may compromise autonomic regulation, particularly parasympathetic function (Table 3).

### 3.6. Individual Results: Most Effective Interventions to Modulate HRV

The analysis identified the three most effective interventions for improving RMSSD, a key marker of vagal-mediated HRV, from the dataset. The top intervention was reported by Toro-Velasco et al. (2009), which demonstrated a substantial RMSSD increase of 66.10, indicating a robust enhancement in parasympathetic activity [40]. The second most effective intervention was from Fernández-Morales et al. (2024), with an RMSSD improvement of 31.10 [29], and the third was from Espejo-Antúnez et al. (2021), showing an RMSSD increase of 21.34 [28]. These findings highlight that those interventions in these studies produced the largest gains in vagal tone, as measured by RMSSD.

### 3.7. Risk of Bias

**Figure 4:**
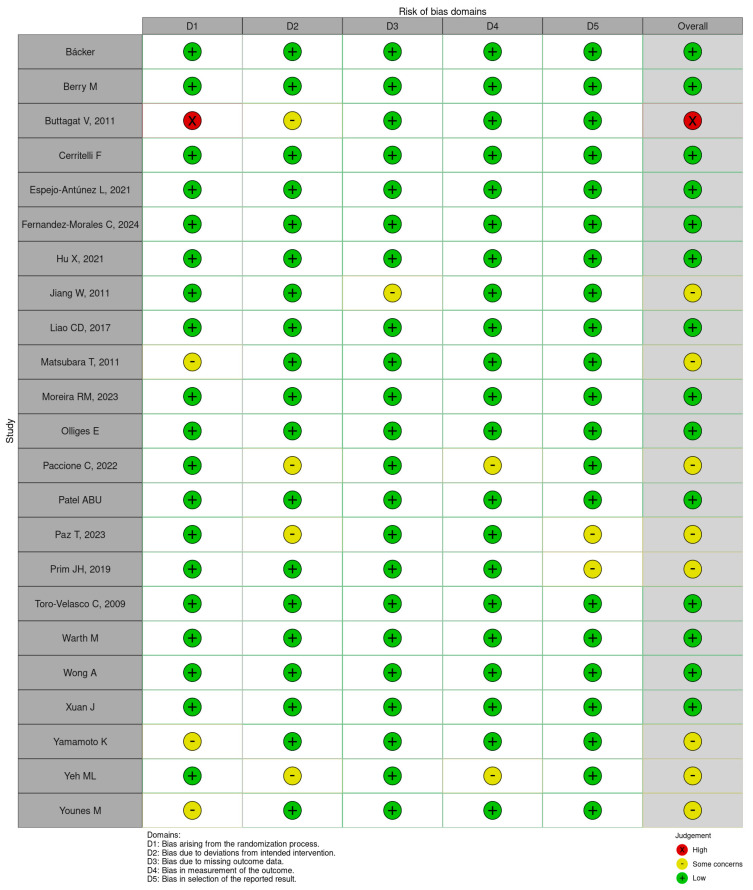
Risk of bias in individual studies across five domains (D1–D5), assessed using the Cochrane Risk of Bias 2.0 tool. Included studies: Bäcker et al. [24], Berry et al. [25], Buttagat et al. [26], Cerritelli et al. [27], Espejo-Antúnez et al. [28], Fernández-Morales et al. [29], Hu et al. [30], Jiang et al. [31], Liao et al. [32], Matsubara et al. [33], Moreira et al. [34], Olliges et al. [35], Paccione et al. [36], Patel et al. [37], Paz et al. [38], Prim et al. [39], Toro-Velasco et al. [40], Warth et al. [41], Wong et al. [42], Xuan et al. [43], Yamamoto et al. [44], Yeh et al. [45], Younes et al. [46].

Out of the 23 included articles, 14 (60.7%) were rated as having a low risk of bias, 8 (34.8%) raised some concerns, and 1 (4.3%) demonstrated a high risk of bias (Figure 4).

## 4. Discussion

In the present meta-analysis of 23 RCTs including 1262 patients, we explored the relationship between changes in HRV metrics with pain treatment. This meta-analysis provides a robust synthesis of randomized controlled trials to evaluate the effect of pain interventions on HRV parameters. Grounded in our original objectives, we structure this discussion by systematically addressing each of the four predefined research questions.

### 4.1. Do HRV Parameters Significantly Differ Between Active and Sham Treatment Groups?

Our findings support a significant improvement in autonomic balance following active pain-modulating interventions, as evidenced by a significant reduction in the LF/HF ratio. While HF power showed a small and non-significant increase across studies, the consistent decrease in LF/HF suggests a relative shift toward enhanced parasympathetic modulation. These shifts show enhanced parasympathetic tone and reduced sympathetic dominance following pain relief, consistent with the restoration of autonomic balance previously documented in pain resolution studies [5,16,17]. The increase in HF power aligns with Porges’ polyvagal theory, which postulates that improved parasympathetic function is associated with reduced threat perception and enhanced regulation of nociceptive signals [47,48].

The lack of significant changes in RMSSD, pNN50, and LF suggests that certain HRV parameters may be more sensitive to detecting autonomic responses to pain interventions than others. A differential responsiveness of HRV metrics has been previously observed by Shaffer and Ginsberg (2017), who demonstrated that frequency-domain measures may better reflect specific autonomic modulation compared to time-domain indices in certain physiological contexts [23]. Additionally, substantial heterogeneity across studies (I^2^ ranging from 44.9% to 82.5%) underscores the variability in study designs, patient populations, intervention modalities, and HRV measurement protocols—all factors known to influence autonomic assessment reliability [49,50].

These findings support the growing recognition that pain and autonomic regulation share overlapping central neural networks, particularly in the anterior cingulate cortex, insula, and periaqueductal gray matter [51,52], regions that simultaneously influence both nociceptive processing and cardiac vagal control.

### 4.2. Are HRV Changes More Pronounced Within the Active Group (Pre- vs. Post-Intervention), Reflecting Both Specific and Non-Specific (Placebo) Effects?

One-group meta-analyses revealed substantially larger effect sizes compared to between-group analyses, with RMSSD showing a large, statistically significant pooled effect and HF power demonstrating a moderate-to-large effect. These robust enhancements in parasympathetic activity post-intervention, regardless of comparator groups, suggest that both specific treatment mechanisms and non-specific factors (e.g., placebo responses, patient expectations, therapeutic alliance) contribute synergistically to autonomic regulation [53,54].

Importantly, SDNN—a measure of overall HRV reflecting total autonomic modulation—also improved significantly (g = 0.455, 95% CI: 0.150 to 0.760; *p* = 0.004), indicating that pain interventions enhance global cardiac regulatory capacity. The LF/HF ratio, however, did not show significant within-group change (g = −0.087, *p* = 0.581), consistent with recent critiques of this metric as an imprecise indicator of sympathovagal balance [55,56]. The inconsistency in LF/HF findings may reflect methodological heterogeneity in measurement protocols, which have been shown to significantly influence frequency-domain analyses [49,50].

These findings highlight the value of within-group analyses in pain research, which capture the cumulative neurophysiological effects of therapeutic context—including expectancy, conditioning, and social learning components that activate endogenous pain modulation systems [57,58]. The larger effect sizes in within-group analyses also align with research demonstrating that contextual factors can amplify autonomic responses through central mechanisms involving prefrontal-brainstem pathways that simultaneously influence both pain perception and cardiovascular regulation [5,51]. This suggests that optimal pain management approaches should deliberately leverage both specific and non-specific mechanisms to maximize autonomic recovery.

### 4.3. Are Specific Types of Interventions More Likely to Modulate HRV?

Among the diverse interventions analyzed, three categories emerged as particularly effective in modulating HRV parameters: manual therapy, electrostimulation, and neuromodulation approaches. Manual therapies (e.g., massage, osteopathic manipulation, and physical therapy) demonstrated consistent improvements in parasympathetic indices, likely through mechanoreceptor stimulation and subsequent activation of periaqueductal gray matter and descending inhibitory pathways [27,59]. Electrostimulation interventions (e.g., interferential therapy, transcutaneous electrical nerve stimulation) showed moderate-to-large effects on RMSSD and HF power, potentially operating through counter-irritation mechanisms and segmental inhibition of nociceptive transmission [60,61]. Neuromodulation techniques (e.g., transcranial direct current stimulation, vagus nerve stimulation) exhibited significant effects on frequency-domain parameters, directly targeting central autonomic networks [24,62,63].

Quantitative analysis identified the three most effective interventions for increasing RMSSD as massage therapy from Toro-Velasco et al. (2009) with a mean increase of 66.10 ms; physical therapy with HRV biofeedback from Fernández-Morales et al. (2024) with an increase of 31.10 ms; and interferential current therapy from Espejo-Antúnez et al. (2021) with an increase of 21.34 ms. These three approaches share a common feature—they provide strong afferent input to the central nervous system, either through mechanoreceptor activation or electrical stimulation, potentially restoring descending inhibitory control over both nociceptive and autonomic circuits [64,65].

The superior efficacy of these interventions may involve several mechanisms: (1) direct stimulation of mechanoreceptors that synapse with inhibitory interneurons in the dorsal horn [60]; (2) activation of descending serotonergic and noradrenergic pathways from the brainstem, modulating both pain and autonomic function [66]; and (3) reduction in central sensitization through normalization of anterior cingulate cortex and insular activity [27,67]. However, the wide heterogeneity in study designs, treatment protocols, and HRV measurement techniques (I^2^ > 70% for most parameters) limits direct comparisons between intervention types. Future head-to-head randomized trials with standardized HRV measurement protocols are warranted to confirm the superiority of specific approaches and elucidate their mechanistic pathways.

### 4.4. Are the Observed HRV Changes Moderated by Markers Such as BMI, Which Could Reflect Systemic Inflammation?

Meta-regression analysis identified BMI as a significant moderator of HRV responses to pain interventions, with pronounced effects on both high-frequency power and LF/HF ratio. These significant moderation effects indicate that patients with higher BMI demonstrated both attenuated improvements in parasympathetic function (lower HF power) and greater sympathetic predominance (higher LF/HF ratio) following treatment. This inverse relationship between BMI and vagal reactivity aligns with established literature linking adiposity to autonomic dysregulation through multiple pathophysiological mechanisms [68,69,70].

The moderation effect of BMI likely reflects underlying metabolic-inflammatory processes relevant to both pain processing and autonomic regulation. Adipose tissue functions as an active endocrine organ, secreting pro-inflammatory cytokines (IL-6, TNF-α) and adipokines that contribute to systemic low-grade inflammation [71,72]. This inflammatory state has been shown to impair vagal function through multiple pathways: direct cytokine effects on brainstem autonomic nuclei [73]; disruption of blood-brain barrier integrity affecting central autonomic networks [74]; and reduced sensitivity of peripheral baroreceptors critical for cardiovascular control [75,76]. We have also shown that increased BMI leads to an attenuation of inhibitory pain processes in osteoarthritis pain [77].

Our findings have important clinical implications, suggesting that patients with elevated BMI may represent a distinct phenotype requiring targeted therapeutic approaches to restore autonomic balance. Previous research has demonstrated that weight reduction interventions improve HRV parameters [69,78], suggesting that metabolic improvements may complement direct pain management. The significant moderation effect of BMI also provides indirect support for the “inflammatory reflex” concept proposed by Tracey (2002), wherein vagus nerve activity regulates systemic inflammation through cholinergic anti-inflammatory pathways [79].

These results underscore the importance of considering individual inflammatory burden when interpreting HRV responses to pain interventions and potentially tailoring treatments based on metabolic profiles. Future clinical trials should explore whether combined interventions targeting both nociceptive pathways and metabolic health (e.g., anti-inflammatory diets, exercise, or pharmacological approaches) yield synergistic benefits in restoring autonomic regulation in patients with pain and elevated BMI [80,81,82].

While our analysis included both acute and chronic pain conditions, the proposed model of autonomic dysregulation as compensatory failure is likely more applicable to chronic pain, where prolonged nociceptive input, central neuroplasticity, and inflammation impact vagal tone. In contrast, autonomic changes in acute pain are typically transient and driven by sympathetic activation. Additionally, the three most effective interventions for improving RMSSD were all conducted in Spanish populations, raising the possibility that cultural or contextual factors—such as therapeutic expectations or clinician-patient dynamics—may have influenced outcomes. These observations underscore the need for future studies to stratify analyses by pain chronicity and explore cultural moderators of autonomic response. Finally, although women comprised over half of the pooled study population, most included RCTs did not report HRV outcomes disaggregated by sex. As such, we were unable to evaluate sex as a potential moderator. Given well-documented sex differences in baseline vagal tone and HRV reactivity, future trials should report sex-specific data to improve the generalizability and mechanistic understanding of autonomic modulation in pain interventions.

## 5. Conclusions

The markedly stronger effects observed in the one-group meta-analyses—particularly in RMSSD and HF—compared to the smaller and often non-significant differences in between-group comparisons, suggest that HRV captures broader physiological shifts beyond the immediate contrast between active and sham interventions. Furthermore, the significant moderating role of BMI on vagally mediated HRV indices (HF and RMSSD) supports the idea that systemic inflammation and metabolic dysfunction influence both pain processing and autonomic regulation.

These findings reinforce the hypothesis that pain and autonomic dysfunction are not strictly causally linked but may instead represent parallel expressions of a shared deregulatory process. In this context, HRV may serve as a critical physiological window into compensatory failure in pain control—a concept aligned with emerging evidence on the compensatory pain system [83]. Rather than reflecting a unidirectional recovery from pain, HRV may help detect individuals whose autonomic and neuroimmune systems are unable to adapt effectively to persistent nociceptive input.

This synthesis highlights HRV’s potential as a clinically actionable biomarker for stratifying patients and tailoring mechanism-based treatments. Future studies should employ standardized HRV protocols and integrate inflammatory and neuroimaging markers to deepen mechanistic understanding and guide personalized interventions at the autonomic-nociceptive interface.

## Figures and Tables

**Table 2:** HRV variables and recording methods considered in the included studies (n = 23).

ID	Author, Year	HRV Parameters and Metrics	Position	Recording Time	IBI Extraction	Software for Analysis
1	Bácker M, 2008 [24]	LF (s^2^), HF (s/L)	At rest	2 min	ECG	Matlab (Mathworks, Natick, MA, USA)
2	Berry M, 2014 [25]	HRV Coherence Ratio (Hz)	At rest	10 min	n/r	n/r
3	Buttagat V, 2021 [26]	LF (ms^2^), HF (ms^2^), LF/HF	n/r	10 min	ECG	n/r
4	Cerritelli F, 2021 [27]	LF (nu), HF (nu), LF/HF	n/r	5 min	Pulse oximeter	Kubios software
5	Espejo-Antúnez L, 2021 [28]	RMSSD	At rest and during intervention	30 min	Firstbeat Bodyguard^®^ monitor	Kubios^®^ HRV software (v.2.1.)
6	Fernández-Morales C, 2024 [29]	LF (ms^2^), HF (ms^2^), LF/HF, RMSSD, pNN50	Prone at rest	15 min	Firstbeat Bodyguard^®^ monitor	Kubios software
7	Hu X, 2021 [30]	LF (ln), HF (ln), LF/HF	n/r	n/r	ZSY-1 Heart Rate Variation Detector, Wegene Technology Inc. (Shenyang, China)	n/r
8	Jiang W, 2011 [31]	RMSSD, SDNN, pNN50, LF (nu), HF (nu), LF/HF	Supine at rest	60 min	ECG	Vivo-VMLA-036-00 3 Logic software
9	Liao CD, 2017 [32]	RMSSD, SDNN, LF (ms^2^), HF (ms^2^), LF/HF	Supine at rest	10 min	ANSWatchVR wrist monitor	ANSWatchVR Manager Pro (version 1.94) software.
10	Matsubara T, 2011 [33]	LF (ms^2^), HF (ms^2^), LF/HF	Supine at rest	5 min	ECG	HRV analysis software (TARAWA/WIN)
11	Moreira RM, 2023 [34]	RMSSD, SDNN, pNN50, LF (ms^2^), HF (ms^2^), LF/HF	Supine at rest	5 min	PolarR RS800CX	Kubios HRV Standard software
12	Olliges E, 2024 [35]	HF (%)	n/r	5 min	ECG	Kubios HRV software version 2.2
13	Paccione CE, 2022 [36]	RMSSD, pNN50, HF (ms^2^)	Seated at rest	3 min	Polar H7	n/r
14	Patel ABU, 2023 [37]	RMSSD, SDNN, LF (ms^2^), HF (ms^2^)	At rest	10 min	ECG	n/r
15	Paz T, 2023 [38]	RMSSD, LF (ms^2^), HF (ms^2^), LF/HF	Supine at rest	10 min	Polar RS800cx	Kubios software, version 2.2
16	Prim JH, 2019 † [39]	RMSSD, SDNN, LF (ms^2^), HF (ms^2^), LF/HF	Seated at rest	2 min	ECG	MATLAB and CardioBatch software
17	Toro-Velasco C, 2009 † [40]	RMSSD, SDNN, LF, HF	Supine at rest	5 min	ECG	NH300 Software
18	Warth M, 2015 [41]	HF (ms^2^)	n/r	5 min	n/r	Kubios software version 2.1
19	Wong A, 2017 [42]	LF (ms^2^), HF (ms^2^), LF/HF	Supine at rest	10 min	Polar device SA-2000E model	n/r
20	Xuan J, 2022 [43]	LF, HF, LF/HF (metrics n/r)	Supine at rest	30 min	ECG	n/r
21	Yamamoto K, 2011 [44]	HF (ms/Hz), LF/HF	Recumbent position	n/r	ECG	Microsoft Excel (2003)
22	Yeh ML, 2018 [45]	LF, HF, LF/HF (metrics n/r)	Sitting position	5 min	HRVm, 8Z11	n/r
23	Younes M, 2017 [46]	RMSSD, LF (ms^2^), HF (ms^2^), LF/HF	Supine at rest	35 min	ECG	SCILAB (INRIA, France).

† Toro-Velasco C, 2009; Abbreviations: RMSSD: Root Mean Square of Successive RR interval Differences; SDNN: Standard Deviation of NN intervals; pNN50: percentage of successive normal-to-normal intervals which exceed 50 ms from previous one; LF: Low frequency; HF: High frequency; LF/HF: Low-frequency/High-frequency ratio; n/r: not reported; ECG: electrocardiogram.

**Table 3:** Meta-regression models exploring the association between HRV outcomes (HF and LF/HF ratio) and moderators, including age, BMI, and pain type.

Model	Moderator	HF			LF/HF Ratio		
		β Coefficient (95%CI)	*p*-Value	R^2^	β Coefficient (95%CI)	*p*-Value	R^2^
**Univariate analysis**	Age	0.118 (−0.012, 0.247)	0.075	63.9	0.013 (−0.010, 0.035)	0.278	0.2
	BMI	−0.088 (−0.173, −0.002)	**0.044**	64.4	0.092 (0.009, 0.0174)	**0.029**	82
	**Type of pain**			62.1			0.0
	Nociplastic vs. Neuropathic	29.08 (−69.96, 128.13)	0.565		1.38 (−0.26, 3.01)	0.098	
	Nociceptive vs. Neuropathic	29.43 (−69.61, 128.48)	0.560		1.479 (0.096, 3.055)	0.0657	

Abbreviations: HF = High-frequency power; LF/HF = Low-frequency/high-frequency ratio; R^2^ = proportion of between-study variance explained.

## Data Availability

Data synthesized in this study are from published trials.
